# Oxidative Stress in the Healthy and Wounded Hepatocyte: A Cellular Organelles Perspective

**DOI:** 10.1155/2016/8327410

**Published:** 2015-12-14

**Authors:** Tommaso Mello, Francesca Zanieri, Elisabetta Ceni, Andrea Galli

**Affiliations:** ^1^Department of Biomedical Clinical and Experimental Sciences “Mario Serio”, University of Florence, 50134 Florence, Italy; ^2^Careggi University Hospital, 50134 Florence, Italy

## Abstract

Accurate control of the cell redox state is mandatory for maintaining the structural integrity and physiological functions. This control is achieved both by a fine-tuned balance between prooxidant and anti-oxidant molecules and by spatial and temporal confinement of the oxidative species. The diverse cellular compartments each, although structurally and functionally related, actively maintain their own redox balance, which is necessary to fulfill specialized tasks. Many fundamental cellular processes such as insulin signaling, cell proliferation and differentiation and cell migration and adhesion, rely on localized changes in the redox state of signal transducers, which is mainly mediated by hydrogen peroxide (H_2_O_2_). Therefore, oxidative stress can also occur long before direct structural damage to cellular components, by disruption of the redox circuits that regulate the cellular organelles homeostasis. The hepatocyte is a systemic hub integrating the whole body metabolic demand, iron homeostasis and detoxification processes, all of which are redox-regulated processes. Imbalance of the hepatocyte's organelles redox homeostasis underlies virtually any liver disease and is a field of intense research activity. This review recapitulates the evolving concept of oxidative stress in the diverse cellular compartments, highlighting the principle mechanisms of oxidative stress occurring in the healthy and wounded hepatocyte.

## 1. Introduction

### 1.1. Redox Homeostasis and Oxidative Stress

Accurate control of the cell redox state, which is mandatory for maintaining the structural integrity and physiological functions, is achieved both by a fine-tuned balance between prooxidant and anti-oxidant molecules and by spatial and temporal confinement of the oxidative species. This tight regulation is mainly achieved by controlling the steady-state production and the subcellular compartmentalization of reactive oxygen (ROS) and reactive nitrogen species (RNS), prooxidant enzymes such as NADH/NAPDH oxidases (NOX) and glutathione peroxidases (Gpx) and that of several antioxidant systems such as reduced/oxidized glutathione (GSH/GSSG), reduced/oxidized cysteine (Cys/CySS), thioredoxin (Trx), peroxiredoxin (Prx), superoxide dismutase (SOD), and catalase.

While it has long been recognized that an imbalance between pro- and anti-oxidants is harmful to cells and is a central mechanism in the development of several pathologies including neurodegeneration, atherosclerosis, diabetes, cancer, and aging, the importance of ROS as second messengers in the cell physiology is a relatively recent acquisition. Indeed, many fundamental cellular processes such as insulin signaling, cell proliferation and differentiation, and cell migration and adhesion, just to name a few, rely on localized changes in the redox state of signal transducers mainly mediated by hydrogen peroxide (H_2_O_2_) [1].

The widespread notion of oxidative stress is that an excessive production of prooxidants or exhaustion of the cellular anti-oxidant defenses can lead to oxidative damage to proteins, nucleic acids, carbohydrates, and lipids, in which radical ROS or RNS are generally thought to play a major role. However, since the activities of many proteins involved in the cellular signaling are regulated by the redox state of their oxidizable thiol residues, which act as redox-sensitive molecular-switches [2], oxidative stress can also occur in the absence of direct structural damage by disruption of the redox circuits that regulate many signaling pathways [3]. Among ROS, hydrogen peroxide is supposed to play a major role either directly or indirectly, in the regulation of the thiol/disulphide redox switches [4], because (i) these reactions typically require a two-electron transfer, (ii) H_2_O_2_ is kinetically restricted and thus can be highly selective in substrate oxidation, and (iii) H_2_O_2_ is generated following growth factor, cytokine, or hormone signaling. However, the detailed molecular mechanisms leading to selective thiol oxidation in redox-sensitive proteins by H_2_O_2_ are still mostly obscure and are the focus of intense research activity. A growing body of data suggests that altered redox signaling precedes and contributes substantially more than direct radical damage to the development of several human pathologies.

The concept of “oxidative stress,” introduced 30 years ago [5], evolved over time from the original oxidative damage to the cell structure and subsequent stress response to include that of alteration of signaling pathways, redox homeostasis, and redox adaptation to stress [6, 7].

Consequently, oxidative stress is not necessarily harmful and antioxidants are not utterly beneficial. In fact, many clinical trials failed to prove the efficacy of low-molecular weight antioxidants in the treatment of several pathologies, and the use of the antioxidants selenium, beta-carotene, and vitamin E was even found to increase overall mortality in a large meta-analysis [8].

Our understanding of the redox landscape of the cell is rapidly evolving and thanks to the recent development of specific redox probes [9–12] we are beginning to unravel a complex spatial and temporal organization of the redox fluxes in the living cells. Compartmentalization of the redox circuitry is crucial to maintain physiology and is a key to understand the alterations of the redox homeostasis occurring in disease.

**Figure 1:**
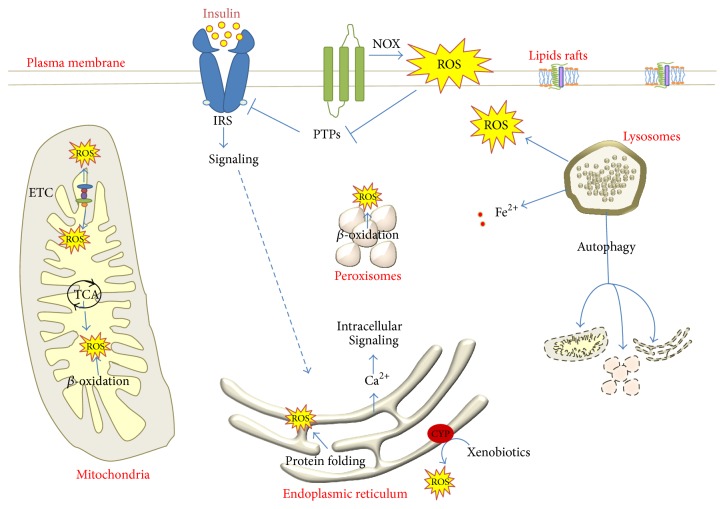
Sites of physiologically produced ROS. Plasma membrane localized ROS bursts deactivate PTPs and allow signal transduction (i.e., by insulin or IGF-1) after tyrosine kinase receptor activation. Mitochondria produce ROS during cellular respiration and metabolic activity. ROS are produced in the ER during protein folding and detoxification by the cytochrome P450 systems. Lysosomes are required for iron metabolism and the removal of damaged cellular components through autophagy. Peroxisomes produce ROS during metabolic or detoxification activities.

**Figure 2:**
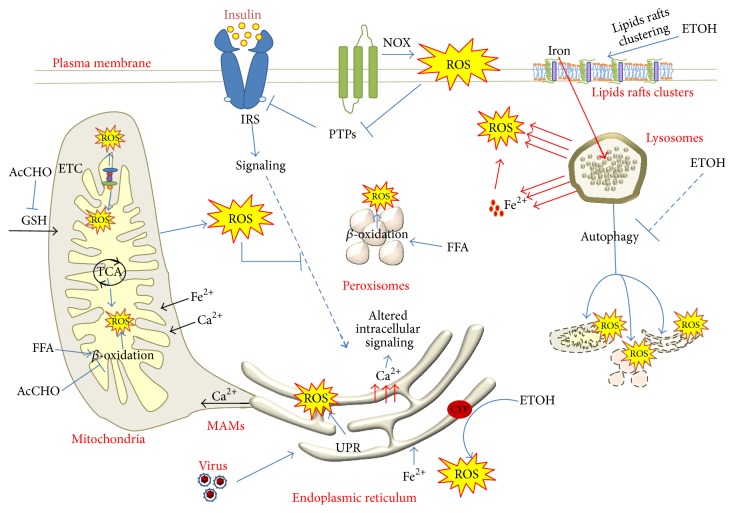
Mechanisms of enhanced ROS production during hepatocyte damage. Ethanol metabolism promotes strong ROS production in the ER by the inducible CYP. It impairs GSH import in the mitochondria, preventing ROS removal. It also impairs *β*-oxidation promoting lipid accumulation. ETOH induces lipid-raft clustering and increases iron uptake, promoting Fe^2+^ leakage from lysosomes and increased Fe^2+^ loads in mitochondria and ER, resulting in ROS production. Ethanol also reduced the autophagic removal of damaged cellular components. Viral infection challenges the ER protein folding process leading to ROS production and Ca^2+^ leakage in the cytosol and mitochondria. Increased MAMs formation promotes Ca^2+^ efflux from ER into mitochondria, increasing mitochondrial ROS production.

The liver is the main metabolic organ and plays a fundamental role in whole body detoxification and blood stream filtering. Most detoxification processes (drugs, alcohol, and endo- and xenobiotics) are carried out through oxidative reactions by the cytochrome P450 (CYP) isoenzymes, which generate superoxide anion (O_2_^∙−^) (Figure 1). Derangement of the liver metabolic processes, such as those occurring by fatty acids overload in NAFLD, results in increased ROS production by increased electron transfer during mitochondrial *β*-oxidation, as well as increased CYP2E1 expression and activity [13]. Strong induction of CYP2E1 also occurs due to excessive consumption of ethanol, whose toxic metabolite acetaldehyde (AcCHO) generates oxidative stress through a number of direct and indirect mechanisms [6, 14, 15] (Figure 2). Hepatotropic viruses cause direct oxidative damage to the endoplasmic reticulum (ER) of hepatocytes, triggering an inflammatory response which propagates the oxidative stress-induced damage to neighboring cells. Activation of the inflammatory signaling pathways in the hepatocyte (i.e., due to translocation of bacterial toxins from the gut or inflammatory cytokines release from visceral fat in obese subjects) is a trait d'union molecular mechanism that synergistically propagates the oxidative stress within different cellular compartments, regardless of the initiating agent.

The liver also plays a central role in heme catabolism and iron recycling, which poses an additional threat since iron catalyzes the formation of ROS through the Fenton and Haber-Weiss reactions (see Section 1.2). Iron accumulation in the liver is associated with increased oxidative stress and can occur as a consequence of genetic disorders (as in hemochromatosis) but also secondarily to other liver diseases, such as NAFLD [16–18] or HCV infection [19, 20], significantly contributing to the progression of disease.

Therefore, the liver has an enormous potential for the generation of oxidative species, and virtually any noxia targeting the liver results in elevated oxidative stress that, when chronic, promotes the fibroproliferative response and progression through fibrosis, cirrhosis, and eventually hepatocellular carcinoma. The onset and progression of chronic liver disease require a complex interplay among different cellular components of the liver, hepatocytes, cholangiocytes, Kupffer cells, sinusoidal endothelial cells, and hepatic stellate cells, mostly orchestrated through a proinflammatory and profibrogenic crosstalk in which oxidative stress mediators such as H_2_O_2_ or nitric oxide (^•^NO) are active players.

As we become more and more aware of the complexity of the redox signaling underlying crucial metabolic regulations, cell fate decision mechanisms, and intercellular communication, it is easy to foresee that the “redox hepatology” field will shape the liver biology research in the next future.

This review recapitulates the evolving concept of oxidative stress in diverse cellular compartments, highlighting the principle mechanisms of oxidative stress occurring in the healthy and wounded hepatocyte.

### 1.2. Mechanism of Reactive Oxygen and Nitrogen Species (RONS) Mediated Toxicity

Several ROS (O_2_^∙−^, ^•^OH, and H_2_O_2_) and RNS (^•^NO, ONOO^−^) are generated inside the cells under physiological and pathological conditions. The biological activity of RONS toward cellular substrate is not equivalent: the hydroxyl radical (^•^OH) has an indiscriminate reactivity toward most biological substrates and is the most relevant ROS involved in oxidative DNA damage while O_2_^∙−^, the most abundant mitochondrial ROS, preferentially reacts with iron-sulfur clusters in target proteins and is effectively converted to H_2_O_2_, which is the principal oxidant of low pKa cysteine residues (Cys) acting as sulfur switches in redox-sensitive proteins [21].

Nitric oxide is a highly diffusible signaling molecule generated by Nitric Oxide Synthases (eNOS, iNOS, and mNOS) in the cytoplasm, extracellular space, and possibly mitochondria [22] and can react with redox-sensitive cysteine residues in proteins forming nitrosothiols, a mechanism of redox sensing analog to hydrogen peroxide (H_2_O_2_) [23]. ^•^NO does not appear to be toxic at physiological concentrations [24] but can readily react with superoxide anion and generate peroxynitrite (ONOO^−^), a spontaneous reaction occurring at such a fast rate that outperforms SOD capability of removing O_2_^∙−^. Peroxynitrite is therefore formed whenever ^•^NO and O_2_^∙−^ are produced simultaneously [25]; it is highly reactive toward iron-sulfur clusters (present in several metabolic enzymes like mitochondrial aconitase and alcohol dehydrogenase), can oxidize protein thiols, and promote tyrosine nitration in target proteins (i.e., Complexes I, II, III, and V of the mitochondrial Electron Transport Chain (ETC)), thereby impairing both the redox- and phosphorylation-dependent cellular signaling. A major mechanism of peroxynitrite toxicity is mediated by lipid peroxidation that causes the degradation of membranes through radical reactions, leading to changes in membrane permeability and fluidity. Finally, oxidative damage to DNA is a hallmark of high-level oxidative stress not only in the nuclei but also in mitochondria [25].

Moreover, while highly reactive radical ROS and RNS are generally diffusion-limited, some ROS and RNS can easily diffuse through biological membranes thanks to their non-/low-polar nature (H_2_O_2_, ^•^NO, and peroxynitrous acid ONOOH) and to dedicated transporter such as aquaporins (for H_2_O_2_) or the HCO_3_^−^/Cl^−^ anion exchanger (for ONOO^−^). The pKa for the couple ONOO/ONOOH is 6.8, very close to physiological pH, thus implying that both mechanisms of peroxynitrite diffusion are relevant in vivo [26]. Therefore, depending on the given RONS involved, oxidative stress can elicit localized alteration of the redox state, localized structural damage, or spread among different cellular compartments and neighboring cells. An elucidating mechanism of such event is the ROS-Induced ROS Release (RIRR) [27], that is, the temporarily opening of mitochondria permeability transition pore (mPTP) that elicit an amplified ROS production after an oxidative challenge. ROS released during RIRR may spread to neighboring mitochondria and, depending on the level of ROS release, either promote mitophagy and removal of nonfunctional mitochondria or trigger a ROS avalanche that can lead to cell death. Of importance, RIRR is considered to be the main mechanism of hepatocyte damage during ischemia/reperfusion injury that occurs following hepatic surgery or transplantation.

Iron overload constitutes a source of oxidative stress of particular relevance in the liver, since hepatocytes and Kupffer cells are the main cell type devoted to iron storage in the body. Iron is an essential component of oxygen sensing proteins, oxygen transport systems, and iron-sulfur containing enzymes [28]; it is a transition metal readily converted between the reduced ferrous (Fe^2+^) and the oxidized ferric (Fe^3+^) forms. The majority of iron in biological complexes is kept as Fe^3+^, while iron reduction to Fe^2+^ is crucial for its mobilization and transport through membranes, loading on ferritin and heme synthesis [28].

In the hepatocyte, iron is stored in the cytoplasm, ER, mitochondria, and lysosomes largely as ferritin-bound Fe^3+^. About 0.2%–5% of the total cellular iron is considered as intracellular transiently mobile “labile pool,” either “free” iron or loosely bound “chelatable” iron, both mainly in the form of redox-active Fe^2+^ [29]. The “labile pool” iron is potentially toxic, since it can catalyze the formation of dangerous ^•^OH radical through the Fenton (1) and Haber-Weiss (3) reactions:

(1) Fe2++H2O2⟶Fe3++OH−+O∙H

(2) Fe3++O2∙−⟶Fe2++O2

(3) The  net  reaction:  O2∙−+H2O2⟶OH−+O2+O∙H

Therefore, leakage of Fe^2+^ from the lysosome due to altered membrane permeability, as well as reduction of Fe^3+^ by superoxide (2), can catalyze the production of ROS and promote lipid peroxidation and severe cellular damage (Figure 2). Mitochondria are particularly susceptible to iron-mediated oxidative stress due to the high production rate of O_2_^∙−^ and its dismutation product H_2_O_2_ during cellular respiration in close proximity to several Fe-S containing enzymes [30].

## 2. Mitochondria

Among cellular organelles, mitochondria account for the largest amount of electron transfer to oxygen thanks to the electron transfer chain (ETC) complexes I–V. ETC complex I (NADH ubiquinone oxidoreductase) and complex II (ubiquinone cytochrome c oxidoreductase), as well as other mitochondrial enzymes such as *α*-ketoglutarate dehydrogenase, pyruvate phosphate dehydrogenase, fatty acyl CoA dehydrogenase, and glycerol 3-phosphate dehydrogenase [31], can produce O_2_^∙−^ as byproduct [32], releasing it within the mitochondrial matrix. Moreover, H_2_O_2_ is produced by the monoamine oxidases (MAOs) located in the outer mitochondrial membrane [33] (Figure 1). Therefore, mitochondria are often referred to as a major ROS production site, although in fact whether high ROS leakage occurs in the mitochondria, at least in a physiological setting, is still highly debated [34, 35]. Estimates of H_2_O_2_ production, as a measure of O_2_^∙−^ leakage, vary from 2% [36] to 0.1-0.2% [31, 32, 34] of total O_2_ consumption and vary largely depending on tissue origin, experimental settings, and the specific substrate fed to the mitochondria. For liver mitochondria, the rate of ROS leakage could be even lower than 0.1% [32]. In fact, since mitochondria are physiologically prone to produce high ROS levels due to the oxidative phosphorylation process, they are also well equipped with a large array of antioxidant systems and radical scavengers, such as Mn-Superoxide Dismutase (Mn-SOD), CuZn-SOD, GSH, glutathione peroxidase, tioredoxin-2, peroxiredoxins, glutaredoxins, and also catalase [37]. Mn-SOD (SOD2) in the mitochondrial matrix readily catalyzes the dismutation of O_2_^∙−^ to H_2_O_2_, which in turn is eliminated by glutathione peroxidase using reduced glutathione (GSH) as hydrogen donor. Oxidized glutathione (GSSG) is then reduced by NADPH-dependent glutathione reductase. Superoxide released in the intermembrane space by the ETC complex III is scavenged by CuZn-SOD (SOD1), followed again by GPx and GSH to eliminate H_2_O_2_. Since GSH is only synthetized in the cytosol [38] and the mitochondrial pool of GSH (mGSH) is replenished by importing GSH produced in the cytoplasm [39], the GSH/GSSG redox state inside the mitochondria is heavily controlled by GSH import through the 2-oxoglutarate carrier and the dicarboxylate carrier [40–43].

Two major enzymatic antioxidant systems collaborate in the mitochondrial matrix: the GSH-dependent glutathione peroxidase and the NADPH-dependent thioredoxin-2 systems, each with specific cofactors.

It must be noted that although abundant, GSH has very limited spontaneous antioxidant activity but very high affinity for GPx. Within the mitochondria, GPx1 [44, 45] and Gpx4 are the most abundant with GPx1 representing over one-third of total GPx activity in the liver [46]. Gpx1 is the major isoform localized both in the mitochondrial matrix and in the intermembrane space and is mainly devoted to H_2_O_2_ detoxification, while GPx4 preferentially reduces lipid peroxides thereby preventing membrane damage to mitochondria [47].

Nevertheless, a number of molecular mechanisms promote mitochondrial ROS overproduction or decreased antioxidant defense under nonphysiological condition [48]. The alteration of the redox homeostasis of mitochondria is well documented in several human pathologies such as NAFLD, viral infection, and toxic events (Figure 2).

Chronic alcohol feeding depletes the mGSH in several animal models [49–51], leading to enhanced ROS production and mitochondrial damage. The mechanism underlying mGSH depletion involves cholesterol accumulation in the inner mitochondrial membrane that results in excess membrane rigidity and impaired GSH carriers functionality, thus disrupting GSH import from the cytosol (Figure 2). In fact, restoring the membrane fluidity, but not increasing cytoplasmic levels of GSH by N-acetylcysteine administration (NAC), recovers mGSH pool and ameliorates liver damage in alcohol-fed rats [45, 50]. The importance of GSH import in the mitochondria can be appreciated considering that several antioxidant systems depend upon mGSH and that the mitochondrial GSH/GSSG redox state is even maintained in a more reduced steady-state redox potential than in the cytoplasm [52], thus requiring energy expenditure for GSH import.

The absence of protective histones, incomplete DNA repair mechanisms, and the close proximity to ROS production site renders mitochondrial DNA (mDNA) sensitive to oxidative damage, increasing the risk of double-strand breaks and somatic mutations with increased ROS production [53]. Indeed, a single dose of alcohol proved effective in inducing massive mitochondrial DNA degradation through a ROS-dependent pathway [54]. The acute degradation of mDNA is then followed by an overshoot of mDNA synthesis as a compensatory mechanism. However, repeated administration of alcohol (binge drinking) accumulated DNA damage and blocked the adaptive response of mDNA resynthesis, resulting in prolonged hepatic mDNA depletion [55]. mDNA encodes 13 proteins involved in the ETC, two rRNA, and all the tRNA necessary for translating the 13 encoded proteins. Mutations in the mDNA therefore may produce dysfunctional ETC complexes, increase ROS production, and expose the mitochondria to new damage in a vicious circle [53]. Indeed, mitochondrial DNA depletion and mutation have been described in patients with alcoholic and nonalcoholic steatohepatitis [56, 57].

Acetaldehyde (AcCHO) produced by ethanol metabolism is readily detoxified by aldehyde dehydrogenase 1 (ALDH1) in the cytosol and by ALDH2 in the mitochondria. Acetaldehyde oxidation to acetate generates NADH and reduces the NAD^+^/NADH ratio, possibly impairing mitochondrial *β*-oxidation which requires NAD^+^ (Figure 2).

Chronic alcohol administration reduces ALDH activity therefore promoting AcCHO accumulation and inducing adduct formation with lipids, proteins, and mDNA [58, 59].

Failure to efficiently remove AcCHO exposes mitochondria to protein, lipid, and DNA adduct formation such as MDA, 4-NHE, and mixed MAA adducts [15]. Moreover, 4-HNE, a lipid peroxidation derivative, can directly inhibit ALDH2, thus promoting AcCHO accumulation in the mitochondrion in a endangering loop [60].

Consistently, the inactivating polymorphism ALDH2^*∗*^2, common in East Asia, confers reduced alcohol tolerance and is associated with increased risk of gastrointestinal cancer. Very recently, by the use of a knock-in mice harboring the ALDH2 (E487K) mutation, Jin and colleagues recapitulated the ALDH2^*∗*^2 human phenotype including intolerance to acute or chronic alcohol administration, impaired clearance of AcCHO, increased DNA damage, and susceptibility to cancer development [61].

Many of the abovementioned findings also apply to the mitochondria of NASH patients, which have altered morphology [62, 63], reduced or mutated mDNA content [57], reduced oxidative phosphorylation [64], and increased ROS production.

However, the molecular mechanisms initiating the mitochondrial dysfunction in NASH are different and originate by an overwhelming induction of mitochondrial *β*-oxidation rather than its inhibition as in ASH. This is consistent with the increased expression of UCP-2 observed in the mitochondria of several obesity and NASH animal models and in the expansion of peroxisomal *β*-oxidation found in humans. The increased electron flux through the ETC produces oxidative stress, which is strongly associated with the severity of NASH (Figure 2).

Depletion of mGSH occurs in NASH animal models, similar to ASH [65], and in NASH patients that have reduced levels of GSH, SOD, and catalase and increased protein oxidation, a hallmark of increased oxidative stress [66]. In principle, targeting oxidative stress is potential therapeutic option for oxidative liver diseases [67]. Of notice, mGSH depletion can affect also the outcome of potential therapeutic antioxidant treatments, such as the use of SOD mimetics in steatohepatitis. Indeed, the use of SOD2 mimetics in a context of mGSH depletion results in increased H_2_O_2_ levels and increases liver injury in animal models of steatohepatitis, highlighting the importance of a combinatory strategy in the targeting of oxidative stress mechanisms [68].

## 3. Endoplasmic Reticulum

Endoplasmic reticulum (ER) is a master intracellular organelle responsible for protein synthesis, folding, modification, and trafficking. In addition, the ER plays a crucial role in calcium homeostasis and in regulating the biosynthesis of steroids, lipids, and carbohydrates [69].

During the folding process, a protein may be oxidized to form disulfide bonds, isomerized to allow polypeptide rearrangement or reduced to allow unfolding and subsequent degradation [70].

The ER lumen has a high ratio of oxidized to reduced glutathione (GSSG/GSH) (145), which creates an oxidizing environment that promotes disulfide bond formation. The electron transport required for this process is driven by a protein pathway that involves two ER-located enzymes: protein disulphide isomerase (PDI) and ER oxidoreductin 1 (ERO1) [71]. PDI directly accepts electrons, leading to the oxidation of cysteine residues and the formation of disulphide bonds. In turn, ERO1 oxidizes PDI through a flavin-dependent reaction and transfers electrons to molecular oxygen as final acceptor. The use of molecular oxygen as the terminal electron recipient leads to the production of ROS, mainly hydrogen peroxide, contributing to cellular oxidative stress [72].

It has been estimated that about 25% of the ROS generated in a cell derive from ER disulfide bond formation during oxidative protein folding, thus making ER the major site of ROS production [73] (Figure 1).

Furthermore, additional oxidative stress can result from the depletion of reduced glutathione that is consumed during the reduction of unstable and improperly formed disulphide bonds [74]. Therefore, an increase in the protein-folding load in the ER can lead to the accumulation of ROS [75]. Cells have evolved several strategies to oppose the ER accumulation of unfolded and misfolded proteins, which are collectively referred to as the UPR (unfolded protein response).

Under normal physiological conditions, the unfolded or misfolded proteins are directed to degradative pathways to restore the ER homeostasis; however, if the unfolded protein production overwhelm the ER buffering capacity, the UPR can activate a cascade of intracellular events resulting in cell death [76, 77]. The UPR is of major importance in hepatocytes, which are rich in ER content and responsible for the synthesis of proteins, cholesterol, bile acids, and phospholipids [78]. And it is characterized by the activation of three distinct signal transduction pathways: the inositol requiring 1 (IRE1) pathway, the protein kinase RNA-like ER kinase (PERK) pathway, and the activating transcription factor 6 (ATF6) pathway. Under nonstressed condition, these three proteins are kept inactive by binding to a chaperone protein, BiP/GRP78, which is the master regulator of the UPR. Under stressed condition (due to, for example, accumulation of misfolded or unfolded proteins, depletion of ER calcium content, or increase of free cholesterol in the ER lumen) BiP/GRP78 dissociates from the UPR transducers resulting in activation of their respective signaling pathways.

Briefly, the activated IRE1*α* removes a 26-bp intron from the XBP1 mRNA, resulting in the production of a spliced XBP1 protein (XBP1s). XBP1s is a transcription factor that regulates the expression of several genes involved in UPR and ER-assisted degradation (ERAD) to help restore ER homeostasis [79]. The IRE1*α*/Xbp1 pathway is also critical for hepatic lipid homeostasis, since it activates the transcription of master adipogenic regulators such as PPAR*γ* and C/EBPs [80]. In addition, IRE1*α* induces the activation of stress kinases, JNK and p38 MAPK, that promote apoptosis [81].

The PERK pathway activates an antioxidant program focused on ATF4 and nuclear factor-erythroid-derived 2- (NF-E2-) related factor 2 (NRF2) [82, 83]. NRF2 is a key player in antioxidant response. After PERK-mediated phosphorylation, NRF2 translocates to the nucleus and activates the transcription of a set of antioxidant and oxidant-detoxifying enzymes, including NAD(P)H-quinone oxidoreductase (NQ01), heme oxygenase 1 (HO1), and glutathione S-transferase (GST) [84, 85]. In addition, NRF2 and ATF4 induce the transcription of genes whose products are involved in the maintenance of glutathione cellular level, the main redox buffer in the cell [82, 83, 86, 87]. The overall antioxidant effect of the PERK pathway is supported by the finding that a potent ER-stress-inducing chemical, tunicamycin, induces only weak accumulation of ROS in wild-type cells, whereas this treatment induces a toxic accumulation of ROS in cells that lack PERK [75].

Dissociation of BiP/GRP78 from ATF6*α* leads to its translocation to the Golgi, where this protein is processed into its active form [88]. The activated ATF6 translocates to the nucleus and functions as a transcription factor, promoting the expression of downstream target genes involved in ER stress including XBP1, GADD153 (also known as CHOP, a proapoptotic transcription factor that plays a critical role in ER stress-mediated apoptosis), and ER chaperones [89, 90]. ATF6*α* is also a regulator of gluconeogenesis [91].

All together, these three pathways mitigate the ER stress by reducing global protein synthesis, increasing misfolded or unfolded protein degradation, and, simultaneously, increasing the specific expression of proteins that help maintaining the protein folding process in the ER lumen as well as ER integrity [92–94].

Under pathological and/or stressful conditions, in which the demand of protein synthesis, folding, and/or repair is increased, the UPR efficiency decreases, resulting in the accumulation of unfolded protein and misfolded proteins and ER damage [93, 95–98].

Moreover, an overactivation of the UPR leads to a sustained activity of ERO1 as well as induction of ERO1*β* expression [99], resulting in an increased H_2_O_2_ production [100] that is found in several liver diseases such as NASH, ASH, and viral infection (Figure 2).

As a first-line response during UPR activation, ER-related PERK pathway attenuates general mRNA translation and activates the Nrf2 transcription factor [71, 83, 92, 101, 102] that translocates to the nucleus and activates antioxidant responsive element-dependent gene expression [71, 75, 92, 93, 101]. However, in NASH, the UPR-induced Nrf2-mediated response is downregulated [75]. Impaired Nrf2 activity is associated with mitochondrial depolarization/dysfunction, as well as increased hepatic free fatty acid levels, fatty liver, and NASH development [102–104]. Moreover, NASH-related accumulation of misfolded proteins, and related unmitigated ER stress, also induces increased ROS production and macromolecules oxidation in the ER lumen through PDI, leading to intracellular depletion of reduced glutathione [71, 72, 105]. Indeed, when oxidized, PDI with ERO1 acts in the oxidative folding of proteins by allowing proper disulfide bond formation. When reduced, PDI breaks and rearranges disulfides in the nascent proteins until the reduced glutathione pool is depleted [71, 73]. Furthermore, both ER stress and oxidative damage prompt calcium leak from the ER, leading to mitochondrial calcium accumulation, which in turn promotes exacerbated mitochondrial ROS production, further amplifying ER stress [72, 104, 106] (Figure 2). It has been recently suggested that elevated levels of palmitic acid would compromise the ER ability to maintain calcium stores, resulting in the stimulation of mitochondrial oxidative metabolism, ROS production, and, ultimately, cellular dysfunction [75]. Therefore, it appears that ER stress may occur earlier than the onset of mitochondrial dysfunction, ROS accumulation, and apoptosis [107, 108]. Moreover, SREBP-1, the master regulator of triglycerides and cholesterol synthesis, is kept inactive at the ER by interaction with insulin induced gene proteins (INSIGs). During ER stress, proteolytic degradation of Insig-1 releases SREBP [96], which is subsequently processed in the Golgi and finally directed to the nuclei where it activates the transcription of the lipogenic program. In turn, excess fatty acids and cholesterol promote ER stress; thus, the reinforced cycle of ER stress, oxidative stress, and lipogenesis-induced lipotoxicity fuels the pathogenesis of NASH [78].

Alcoholic liver disease (ALD) is certainly related to an excessive production of ROS from ethanol metabolism and the consequent oxidative stress within the hepatocytes [109, 110]. Two metabolic pathways are involved in the degradation of ethanol. First, ethanol is oxidized into acetaldehyde by alcohol dehydrogenase (ADH), followed by production of acetate by means of acetaldehyde dehydrogenase (ALDH). Acetaldehyde mediates most of the toxic effect of alcohol [15, 111, 112]. The second pathway of ethanol degradation, which is largely inducible, operates through the microsomal ethanol-oxidizing system (MEOS) cytochrome P450. CYP2E1, the main cytochrome P450 isoform induced by ethanol consumption, is located at the membrane of ER [113–116], making it the master mechanism of ER ethanol-induced ROS production. Ethanol oxidation by CYP2E1 generates O_2_^∙−^ and H_2_O_2_ promoting membrane lipoperoxidation. Moreover, ethanol administration and ROS production increase free iron, which catalyzes the production of strong oxidants, such as hydroxyl radical (OH^•^), ferrous oxide (FeO), and hydroxyethyl radical (CH_3_CHOH). This damaging mechanism is also common to lysosomes and mitochondria (Figure 2).

The UPR overactivation and ROS production occur also in Hepatitis C and B, but the process that induces these responses is different from other liver diseases.

Hepatitis C virus (HCV) replication in infected host cells is dependent on several viral proteins that are folded in the ER and synthesized in ribonucleoprotein complexes in association with the ER [117]. HCV replication has been shown to cause ER stress and its gene products such as Core, E2, NS5A, and NS4B have also been demonstrated to induce UPR and ROS production [118, 119].

The protein aggregates that are formed in the viral replication induce all three different pathways of UPR which sustain viral replication alleviating ER stress [118, 119]. ROS production during HCV infection also occurs due to altered intracellular Ca^+2^ homeostasis. For example, Core viral protein perturbs the intracellular calcium both by inducing ER Ca^2+^ release and by stimulating the Ca^2+^ uniporter in mitochondria, increasing ROS production in mitochondria and opening of the mPTP [120]. Similarly, NS5A perturbs Ca^2+^ signaling and elevates ROS production in mitochondria leading to activation of transcription factors such as NF-*κ*B and STAT that are involved in HCV-mediated hepatocarcinogenesis [121]. NF-*κ*B activation is also stimulated by NS4B, a mechanism requiring Ca^2+^-induced ROS production [122].

Similar mechanisms of UPR activation and ROS production also occur in HBV infection [123] (Figure 2).

## 4. Lysosomes

Autophagy is a fundamental mechanism of cell adaptation to stress, allowing removal of damaged molecules and cellular components by degradation in the lysosomal compartment, which is of particular importance for the removal of nonfunctional mitochondria (mitophagy) (Figure 1). Alterations of the autophagic pathway play a major role in the onset and perpetuation of several chronic diseases, including neurodegenerative and metabolic disorders as well as cancer chemoresistance. Most of the processes associated with RONS production also stimulate autophagy [124]. For instance, starvation, through inhibition of mTOR pathway, stimulates autophagy and increases mitochondrial ROS production. H_2_O_2_ oxidizes a redox-sensitive thiol of Atg4, which then promotes LC3-I conversion to LC3-II and maturation of the autophagosome [125]. Consistently, autophagy is stimulated in vivo by mitochondrial superoxide production, as seen by experimental downregulation of MnSOD, which increases O_2_^∙−^ and reduces H_2_O_2_ [126]. However, it is not clear whether O_2_^∙−^ directly stimulates autophagy or more likely induces lipid peroxidation and mitochondrial damage which in turn activate autophagy. This second line of thought is supported by the observation that, in nutrient-deprived hepatocytes, mitochondrial membrane depolarization precedes the formation of the autophagosome [127] and defective mitophagy results in accumulation of dysfunctional mitochondria, increases oxidative stress, and promotes tissue liver damage and cancer [128]. Chronic ethanol feeding is associated with decreased intralysosomal hydrolases content and reduced proteasomal activity due to impaired cathepsin L trafficking in the lysosome [129, 130]. Oxidative stress can also harm lysosomal membranes resulting in elevated cytosolic levels of cathepsin B due to lysosomal leakage [131].

The effect of ethanol metabolism on autophagy is quite debated, and controversial results have been reported [132]. Using LC3-GFP transgenic mice, two groups described that binge- [133], acute- or chronic-ethanol administration [134] promoted autophagosome formation in vivo. The increase in autophagosome formation was paralleled by inhibition of mTORC1 signaling pathway [133].

In contrast, using a CYP2E1 knock-out or knock-in mice and parallel in vitro model, Wu and colleagues showed that binge ethanol administration reduced macroautophagy in CYP2E1 KI mice and cells but not in KO mice [135]. Despite the different autophagy status, which could be due to the use of different transgenic models and different binge drinking protocols, inhibition of autophagy enhanced ethanol toxicity while pharmacological promotion of macroautophagy by carbamazepine, as well as rapamycin, was shown to have a therapeutic potential in animal models of acute ethanol-induced toxicity [133] as well as ASH [135] and NASH [136].

Since ethanol is known to reduce the proteolytic activity of lysosomes, it is possible that the degradative part of autophagy, that is, the autophagolysosome formation, may be impaired in chronic ethanol feeding despite increased autophagosome formation, leading to defective removal of damaged cellular components [137] (Figure 2).

This mechanism could be relevant also for the pathogenesis of NAFLD. In vitro experiments showed that autophagy was increased in hepatoma cells exposed to FFA, mimicking the “first hit” of NASH pathogenesis. However, after a “second hit” of H_2_O_2_ or TNF*α* (oxidative damage and inflammation), the autophagic flux diminished despite enhanced autophagosome formation [138].

An important mechanism of ROS induced liver injury is mediated by the release of “labile” iron (Fe^2+^) that occurs in damaged lysosomes during ischemia/reperfusion [139].

Lysosomes are a major storage site of iron, which enters the hepatocyte through transferrin mediated endocytosis, subsequent endosome acidification, iron reduction, and release from transferrin. Fe^2+^ is then released in the cytosol or delivered to mitochondria in controlled amount (Figure 1). Moreover, lysosomes can significantly increase their iron content by reparative autophagic uptake of damaged mitochondria, peroxisomes, or cytosolic iron-loaded ferritin [29]. Labile iron is transported to mitochondria, where it catalyzes the Fenton reaction with H_2_O_2_  (see Section 1.2), producing the deleterious hydroxyl (OH^•^) and hydroxyperoxyl (OOH^•^) radicals which damage mitochondria membranes and induce the opening of the permeability transition pore, triggering the RIRR response and eventually cell death (Figure 2). Fe^2+^ induced ROS formation can represent a potent mechanism of damage amplification not only within the cellular compartments but also at the cell and organ level. In fact, many human diseases, especially liver diseases, are associated with increased serum ferritin levels that arise due to hepatocyte death. Increased ferritin uptake in hepatocytes is associated with abnormal endosome clustering and induces lysosomal membrane permeability and promotes lipid peroxidation, depletion of GSH, and reduction of GSH/GSSG ratio. Ferritin accumulation triggers macroautophagy which is abolished by Fe chelation, confirming the mechanistic role of Fe-induced ROS formation in the onset of ferritin toxicity. Also in this model, pharmacological inhibition of macroautophagy strongly enhanced ferritin toxicity, further substantiating the concept that induction of autophagy is a generalized defense mechanism against ROS-mediated cellular damage [140].

## 5. Peroxisomes

Peroxisomes are ubiquitous organelles involved in catabolic oxidative reactions, xenobiotic detoxification, and bile salt synthesis. In mammals, peroxisomes carry on the *α*-oxidation of very-long chain fatty acids that cannot directly enter the mitochondria for *β*-oxidation[141].

Several peroxisomal oxidases produce ROS, primarily H_2_O_2_, but also ^•^NO, since iNOS was detected in peroxisomes of hepatocytes [142]. Peroxisomes are not only a source of ROS but also a powerful ROS disposal compartment, thanks to a large array of antioxidant enzymes, mainly catalase, but also GPx, Mn-SOD, and CuZn-SOD, and peroxyredoxin-1 and peroxyredoxin-5 [143] (Figure 1).

Peroxisomes are highly dynamic structures that undergo enlargement, elongation, and increase in number in response to several xenobiotic or physiologic stimuli.

Peroxisomes can support mitochondria in the *β*-oxidation of fatty acids [144], a process mediated by the activation of the nuclear receptor PPAR*α* [145]. Accordingly, peroxisomes are elevated in several mice models of NAFLD [146] and proliferation and enlargement of peroxisomes are also described in patients with fatty liver [147] (Figure 2).

Activation of PPAR*α* and subsequent induction of peroxisomal *β*-oxidation is a potent inducer of H_2_O_2_ production which is not paralleled by an equal increase in antioxidant enzymes, mainly catalase, leading to oxidative stress and substantially contributing to the hepatocarcinogenic effects of PPAR*α* activators observed in mice, but not in humans [148].

Disruption of the peroxisomal structure and function in the hepatocyte, as in the PDX5 knock-out mice, resulted in structural alteration and reduced functionality of mitochondria, increased proliferation of ER and lysosomes, and accumulation of lipid droplets. Surprisingly, no sign of significant oxidative stress was found, although metabolic rearrangement toward a more glycolytic setting was observed [149].

## 6. Plasma Membrane

At the plasma membrane interface, key signal transduction events originate following the ligand-receptor interaction. Signal transduction is biologically encoded by phosphorylation of key Tyr or Ser/Thr residues that induce conformational and functional changes in transducing proteins. The coordinated activities of protein kinases and protein phosphatases ensure the substrate, temporal, and spatial confinement required to obtain specificity for signal transduction events. Signal transduction occurs not only by means of the well-known “phosphorylation code,” but also by regulation of redox-sensitive Cys residues in signaling proteins. Such “redox-code” [21] adds a level of complexity and flexibility to signaling circuitry.

The best characterized redox regulation of kinase signaling, and likely the most relevant to the hepatocyte, is the insulin pathway. Insulin binding to its receptor activates intrinsic kinase activity and initiates a phosphorylation cascade through the PI3K/AKT/mTOR pathway. In parallel, insulin promotes the deactivation of protein phosphatases (PTEN, PP2A, and SH2P), a mechanism requiring localized H_2_O_2_ burst at the plasma membrane generated by extracellular NOX [150–153] (Figure 1).

Adiponectin, which potentiates insulin action, activates 5-LOX H_2_O_2_ bursts and deactivates PTP1B [154].

Exogenous H_2_O_2_ administration can therefore modulate insulin signaling through inhibition of Protein Tyrosine Phosphatases (PTP), as recently demonstrated by Iwakami et al. [155].

The ROS-mediated inactivation of PTP is a general physiological mechanism necessary for efficient signal transduction of several, if not all, MAPK family members [2], including p38 [9, 156], JNK [157], and ERK1/2 [158] (Figure 1).

Removal of signaling H_2_O_2_ and reduction of oxidized Cys in PTP are ensured by cytoplasmic antioxidants, such as GSH, thioredoxin 1 [159] and peroxiredoxin-2 [152], thus terminating the signaling activity.

Although H_2_O_2_ is required for normal signal transduction, excess oxidative stress can activate stress-sensitive Ser/Thr kinase such as JNK, ERK1 and IKKb (inhibitor of NF-*κ*B), and p70S6k, all of which are client of the redox-regulated protein phosphatase PP2A. Stress sensitive kinases take part in insulin resistance by inhibitory phosphorylation of IRS-1 [160] (Figure 2).

Clearly, redox signaling is highly intertwined with phosphorylation signaling, and a high level of spatial and temporal confinement must be fulfilled to ensure the proper biological response.

Very recently, Hua and colleagues uncovered the coordinated redox mechanisms initiating and terminating hepatocyte proliferation during liver ontogenesis or regeneration after partial hepatectomy [161]. They found that sustained elevated H_2_O_2_ levels are required for the activation of ERK signaling and trigger a shift from quiescence to proliferation, while sustained decreased H_2_O_2_ levels activate p38 signaling and trigger a shift from proliferation to quiescence. Pharmacological lowering of H_2_O_2_ levels reduces the extent of fetal hepatocyte proliferation and delays the onset of liver regeneration. Chemical augmentation of H_2_O_2_ levels in adult hepatocytes triggers proliferation and delays the termination of liver regeneration. Although the authors did not map the precise cellular location of H_2_O_2_ production, the mechanism triggering the H_2_O_2_-induced hepatocyte proliferation during embryogenesis and liver regeneration involves early induction of NADPH Oxidase 4 (NOX4) and the adaptor protein p66shc [161], which is known to be necessary for NOX assembly at the plasma membrane [162]. Interestingly, NOX4 recruitment at the plasma membrane and localized H_2_O_2_ production are also required for IGF-I signal transduction to Src, a mechanism mediated by the scaffold protein SHPS-1 [163], suggesting that NOX4 assembly at the plasma membrane could promote specific signal transduction by redox regulation of different client effectors.

In hepatocytes, membrane fluidity and lipid-raft clustering have been described to mediate ethanol-induced oxidative stress. ROS produced by ethanol metabolism increase plasma membrane fluidity and determine an increase in low-molecular weight iron, triggering massive ROS production and membrane peroxidation [164]. These event are associated with lipid-raft clustering, intraraft disulfide-bond and peroxidation, phospholipase C recruitment inside the lipid-raft, and activation of a signaling pathway that modulate iron content in the lysosomal compartment [165] (Figure 2). Consistently, reducing lipid-raft clustering and oxidation by DHA administration [166] or increasing plasma membrane fluidity by benzo(a)pyrene administration [167], respectively, alleviated or worsened the ethanol toxicity mediated by increased lysosomal iron leakage. Since membrane lipid rafts are required for peroxisome biogenesis [168], it would be interesting to assess the effect of ethanol-induced lipid-raft clustering on peroxisome structure and function.

## 7. Concluding Remarks

Redox homeostasis in the cell is achieved by accurate compartmentalization of prooxidants and RONS buffering systems. Each cellular compartment differs in the overall redox state as well as in the concentration and oxidative steady-state of its antioxidant pairs. Lysosomes and ER are relatively more oxidizing than cytoplasm, while more reducing conditions are found in the nuclei and mitochondria. Each of these compartments is actively maintained in a nonequilibrium redox state to fulfill specific tasks. At the same time, cellular organelles are highly interconnected by a complex network of physical connections, signaling pathways, intracellular trafficking routes, and metabolic activities.

Oxidative stress can arise by different mechanism in any cellular compartment and spread to other cellular organelles through any of the physical or functional connections.

For instance, mild alterations of plasma membrane permeability induced by ethanol metabolism promote clustering of lipid raft and increase ferritin uptake that promote membrane lipid peroxidation and free iron leakage from the lysosome. Free iron is eventually transported to the mitochondria where it triggers a burst of oxidative stress and opening of the mPTP, propagating the oxidative damage through the RIRR mechanism (Figure 2).

Perturbation of the network of physical and functional connections between cellular organelles is not only a consequence of pathological oxidative stress but can be indeed the primary causal mechanism of oxidative stress and morbidity. In a recent paper, Arruda and colleagues, by using an elegant in vivo strategy, demonstrated that artificially increasing the physical interaction between mitochondria and ER (MAMs: mitochondrial-associated ER membranes) in the liver led to altered calcium load in the mitochondria, increased oxidative stress, and metabolic alterations, promoting obesity and metabolic dysfunction [169]. Rearrangement of ER around mitochondria seems to be an early event after HFD treatment and likely reflects a transient adaptation of this physiologically highly dynamic process (Figure 2).

The recent development of genetically encoded ROS sensor paved the way for a new era of exploration in the redox cell biology, allowing real-time detection and measure of the redox dynamics in living cells. These technological advances are contributing to unravel the complex spatial and temporal dynamics of H_2_O_2_ bursts that, acting as switches, can activate or deactivate signaling components, thus interconnecting the redox-based and kinase-based signaling pathways.

As we deepen the understanding of the redox homeostasis in the different cellular compartment, the concept of “oxidative stress” evolves and alterations of redox mechanisms are increasingly being recognized in human diseases, revealing the potential for new therapeutic opportunities.

## Abbreviations

IRS: Insulin receptor substrate
NOX: NADPH Oxidase
PTPs: Protein Tyrosine Phosphatases
ETC: Electron Transport Chain
TCA: Tricarboxylic acid cycle
FFA: Free fatty acids
GSH: Reduced Glutathione
CYP: Cytochrome P450 system
UPR: Unfolded protein response
MAMs: Mitochondria associated membranes
ETOH: Ethanol
AcCHO: Acetaldehyde.
